# *Acacia Holosericea*: An Invasive Species for Bio-char, Bio-oil, and Biogas Production

**DOI:** 10.3390/bioengineering6020033

**Published:** 2019-04-16

**Authors:** Md Sumon Reza, Ashfaq Ahmed, Wahyu Caesarendra, Muhammad S. Abu Bakar, Shahriar Shams, R. Saidur, Navid Aslfattahi, Abul K. Azad

**Affiliations:** 1 Faculty of Integrated Technologies, Universiti Brunei Darussalam, JalanTungku Link, Gadong, BE 1410, Brunei Darussalam; 18h0251@ubd.edu.bn (M.S.R.); 15h1553@ubd.edu.bn (A.A.); wahyu.caesarendra@ubd.edu.bn (W.C.); saifullah.bakar@ubd.edu.bn (M.S.A.B.); 2 Department of Chemical Engineering COMSATS University Islamabad Pakistan, Lahore Campus, Raiwind Road Lahore, Punjab 54000, Pakistan; 3 Mechanical Engineering Department, Faculty of Engineering, Diponegoro University, Jl. Prof. Soedharto SH, Tembalang, Semarang 50275, Indonesia; 4 Civil Engineering Programme Area, Universiti Teknologi Brunei, Jalan Tungku Link, Gadong BE 1410, Brunei Darussalam; shams.shahriar@utb.edu.bn; 5 Research Center for Nano-Materials and Energy Technology (RCNMET), School of Science and Technology, Sunway University, Bandar Sunway, Petaling Jaya, Selangor Darul Ehsan 47500, Malaysia; saidur@sunway.edu.my; 6 American University of Ras Al Khaimah, Ras Al Khaimah, PO Box 10021, UAE; 7 Department of Mechanical Engineering, Faculty of Engineering, University of Malaya, Kuala Lumpur 50603, Malaysia; navid.fth87@yahoo.com

**Keywords:** invasive species, *Acacia Holosericea*, biomass, bio-char, bio-oil, biogas

## Abstract

To evaluate the possibilities for biofuel and bioenergy production *Acacia Holosericea*, which is an invasive plant available in Brunei Darussalam, was investigated. Proximate analysis of *Acacia Holosericea* shows that the moisture content, volatile matters, fixed carbon, and ash contents were 9.56%, 65.12%, 21.21%, and 3.91%, respectively. Ultimate analysis shows carbon, hydrogen, and nitrogen as 44.03%, 5.67%, and 0.25%, respectively. The thermogravimetric analysis (TGA) results have shown that maximum weight loss occurred for this biomass at 357 °C for pyrolysis and 287 °C for combustion conditions. Low moisture content (<10%), high hydrogen content, and higher heating value (about 18.13 MJ/kg) makes this species a potential biomass. The production of bio-char, bio-oil, and biogas from *Acacia Holosericea* was found 34.45%, 32.56%, 33.09% for 500 °C with a heating rate 5 °C/min and 25.81%, 37.61%, 36.58% with a heating rate 10 °C/min, respectively, in this research. From Fourier transform infrared (FTIR) spectroscopy it was shown that a strong C–H, C–O, and C=C bond exists in the bio-char of the sample.

## 1. Introduction

The rapid growth of invasive plants is a severe threat to natural ecosystems throughout the world as well as the danger to public health and economies [1,2]. To overcome this threat, mechanical (pulling and digging) and chemical (herbicides) control methods are mainly practiced which require huge capital and effort with little return [3]. The massive volume of biomass from invasive plants create a big challenge for disposal as well. It has been shown that piling or dispersing cut portions of invasive species can result in the restoration by new plants from propagule pieces [4]. Therefore, it is essential to develop innovative and cost-effective policy to control invasive plants. Modern developments in biofuel technology envision a new approach to manage invasive plants by converting them into value-added products, such as bio-char and bioenergy [5]. 

Biofuel technology has gained increasing attention recently because of its ability to reduce global warming caused by emissions of carbon dioxide (CO_2_) and greenhouse gasses. From 2000, CO_2_ emissions have gone up by more than 3% annually which is risky and irreversible [6]. A mitigation policy that draws down surplus CO_2_ from the atmosphere would then assume an importance greater than an equivalent diminution in emissions [7]. Bio-char production and its storage in soils, has been recommended as one of the possible measure for reducing the atmospheric CO_2_ concentration [8]. For environmental protection, the utilization of bio-char as activated carbon has a strong control on water and air purification, vehicle exhaust emission, solvent recovery, and catalyst support because of its high pore surface area, sufficient pore size distribution, and high mechanical strength [9].

A range of pyrolysis technologies are available that yield different proportions of bio-char, bio-oil, and biogas. Biogases are typically used to generate electricity; bio-oil may be used directly for low-grade heating applications or as a diesel substitute after treatment [10]. Co-gasification of Acacia with 40% polyethylene terephthalate (PET) plastic waste can produce a good amount of hydrogen (~52%), but output gases are not desirable [11]. Very few researches have been done to estimate the production of biofuel from invasive plants [12]. Pyrolysis processes are divided into two major types, fast and slow, which refer to the rate at which the biomass is changed. Fast pyrolysis, with biomass residence times of a few seconds, generates more bio-oil and less bio-char. On the other hand, slow pyrolysis, for which biomass residence times can be hours to days, produces more bio-char and less bio-oil [13,14,15].

Among all the invasive plants, Acacia species are well known for their invasiveness and negative effects on native trees, causing serious threats to the biodiversity [16,17]. Figure S1 shows the regions of the total 1350 different species of Acacia in the world, which can grow in warm, tropical, and even deserts. Acacia trees exhibit high biomass production within a few years because of their fast growth and can be utilized as a sustainable resource for biofuel production by minimizing these invasive plants [18,19]. 

There are four types of Acacia species in Brunei Darussalam namely *Acacia Mangium, Acacia Auriculiformis, Acacia Cincinnata,* and *Acacia Holosericea* [17]. According to the Pacific Island Ecosystems at Risk (PIER), the risk scores are 8, 13, 7, and 4 for the above four species, respectively [20]. It is recommended to assess the viability and commercial feasibility of using Acacia species as an alternative source of renewable energy to achieve Brunei’s aims in dropping CO_2_ emissions while also ensuring the country’s biodiversity is not harmfully impacted by these invasive species [2]. Among the species, *Acacia Holosericea* is an important invasive tree in this region [21] as shown in Figure S2. The objective of this research is to develop an innovative strategy to produce value-added bioenergy from the *Acacia Holosericea* species. In this work, we evaluate invasive plants as feedstock to study the bio-energy production capacities. The results generated in this study may lead to scale-up actions aimed at the use of invasive biomass for the production of biofuels or fine chemicals. 

## 2. Experimental

### 2.1. Material Preparation

The samples were cut into small pieces from *Acacia Holosericea* trees (collected from Universiti Brunei Darussalam campus), mainly the trunk part. These pieces were placed under direct sunlight for 4 weeks to eliminate the moisture content. After that, the samples were crushed in a hammer mill to get small particles. The crushed particles were sieved by regular sieves to obtain uniform particle size less than 0.25 mm. Finally, the biomass samples were stored in airtight bags to avoid contact with air. As *Acacia Holosericea* is a shrub with a maximum of 25 cm tall, this preparation process is economical rather than the literature of *Acacia Auriculiformis and Acacia Mangium* which are around 300 cm tall [2,14,22]. In addition, some of the researches used an electric oven for drying the sample, whereas, only sunlight was used in this work [22,23]. The sample preparation diagram is shown in Figure S3.

### 2.2. Sample Tests and Analysis

**Figure 1:**
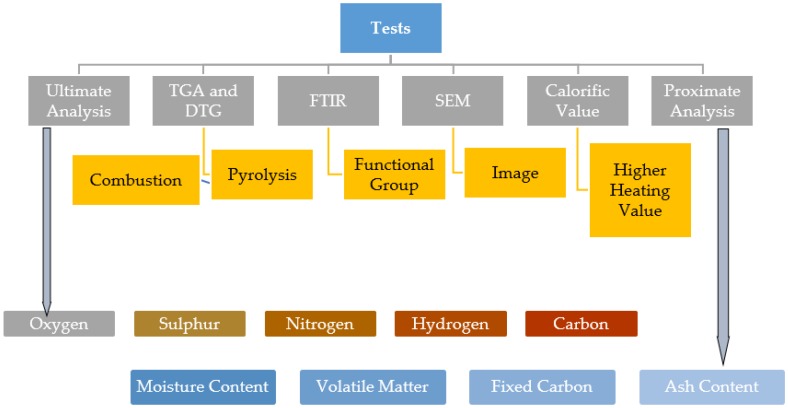
Analysis diagram for the characterization of the *Acacia Holosericea* sample.

In Figure 1, the schematic diagram of the test performed for the *Acacia Holosericea* sample is shown.

#### 2.2.1. Proximate Analysis 

As a source of energy, for any type of biomass, proximate analysis is one of the most important methods for measuring the properties, such as moisture content, volatile matter, and ash content. The standards and procedures of the American Society of Testing Materials (ASTM) were followed for analyzing the percentages of moisture content (MC), volatile matters (VM), and ash content (AC). The percentage of fixed carbon was measured by deducting the total percentage of MC, VM, and AC from 100.

The ASTM D 3173-87 method was used for moisture content analysis. All analyses were performed twice for every test, and the average results were considered. One gram of *Acacia Holosericea* sample was measured by preheated crucible. Then, the sample with crucible was placed in an oven for 3 hours maintaining a constant temperature of 105 °C. The percentage of moisture content (MC) was calculated by the following Equation (1):

(1) $$\mathrm{Moisture}\;\mathrm{content}\;\left(\mathrm{MC}\right)\;\left(\mathrm{wt}\%\right)=\frac{wcs_{i}-wcs_{f}}{ws_{i}}\times 100$$

where,

*ws_i_* is the original weight of the biomass sample,
*wcs_i_* is the initial weight of the sample with crucible (before heating), and
*wcs_f_* shows the final weight of the sample with crucible (after heating).

The ASTM D 3175-07 method was used for measuring the percentage of volatile matter (VM) in biomass. VM was calculated by two steps. First, weight losses of the sample were determined by Equations (2) and (3).

(2) $$\mathrm{Weight}\;\mathrm{Loss}\;\left(\mathrm{wt}\%\right)=\frac{wms_{i}-wms_{f}}{ws_{i}}\times 100$$

where,

*ws_i_* is the original weight of the biomass sample,
*wms_i_* is the initial weight of the biomass sample with crucible (before drying), and
*wms_f_* is the final weight of that sample and crucible (after drying.)

Volatile matters (VM) (wt%) = Weight loss (wt%) − Moisture Contents (wt%)(3)

For Equation (3), weight loss (%) was calculated from Equation (2), and the moisture contents (%) were calculated from Equation (1). 

For Ash contents, the ASTM D 3174-04 method was used using the following Equation (4):

(4) $$\mathrm{Ash}\;\mathrm{Contents}\;\left(\mathrm{wt}\%\right)=100-\frac{wds_{i}-wds_{f}}{ws_{i}}\times 100$$

where

*ws_i_* is the original weight of the sample,
*wds_i_* is the preliminary weight of the sample and crucible (before drying), and
*wds_f_* is the final weight of the biomass sample with crucible (after drying)

The percentage of fixed carbon (FC) was determined by Equation (5).

Fixed carbon (FC) (wt%) = 100 − (MC% + VM% + AC%)(5)

#### 2.2.2. Ultimate Analysis 

To obtain the result of the ultimate analysis of *Acacia Holosericea* samples, a carbon, hydrogen, nitrogen and sulphur (CHNS) analyzer, EA 1112 Series, manufactured by Thermo Quest, Italy, was used by the total oxidation method. The percentage of carbon (C%), nitrogen (N%), hydrogen (H%) and sulphur (S%) was calculated. Percentage of oxygen was determined by deducting the total percentage of CHNS from 100 by the following Equation (6):

Oxygen contents (wt%) = 100 − (C% + H% + N% + S%)(6)

#### 2.2.3. Calorific Value Analysis 

To determine the higher heating values (HHVs), calorific value analysis of the *Acacia Holosericea* biomass sample was carried out by the bomb calorimeter (C-200) manufactured by PA Hilton of UK, by following the ASTM D 5468–02 procedure.

#### 2.2.4. Thermogravimetric Analysis (TGA and DTG)

For thermogravimetric analysis (TGA) and their first derivative (DTG), A Perkin Elmer Thermogravimetric analyzer (TGA7) was used to examine the thermochemical performance under the condition of combustion and pyrolysis. The TGA and DTG analysis of the biomass sample was performed for temperatures from 40 to 900 °C with a heating rate of 25 °C/min. In pyrolysis, pure nitrogen (N_2_) gas was supplied continuously in the heating chamber for maintaining the inert atmosphere. Similarly, for combustion, pure oxygen (O_2_) gas was delivered continuously to the heating chamber. The curve for TGA and DTG was analyzed to determine the degradation stages of the biomass sample.

#### 2.2.5. Pyrolysis

**Figure 2:**
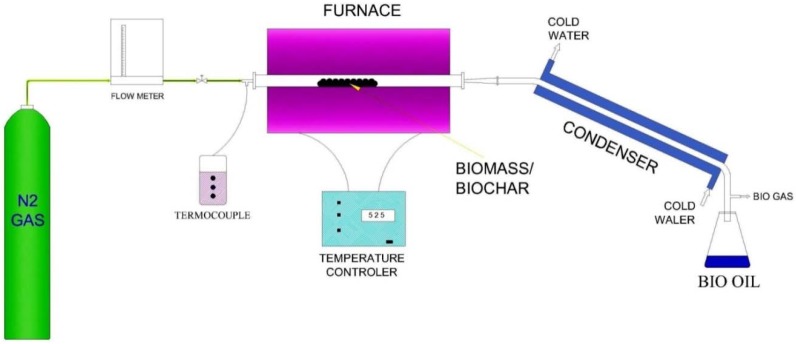
Schematic diagram of the pyrolysis set up.

The pyrolysis of the *Acacia Holosericea* sample was done by a stainless steel fixed bed reactor (2.7 cm inner diameter and 50 cm length) inserted in a horizontal tube furnace made by Carbolite Gero 300–3000 Electrical Furnaces. The reactor was filled with a 30 g of sample. For this work, Pyrolysis was performed for deriving bio-char and bio-oil at two temperatures, 500 °C and 600  °C, with a heating rate of 5  °C/min and 10 °C/min, respectively, with a constant nitrogen flow rate of 0.5 L/min. The process continued for 60 min after the final temperature was obtained. The products of char located inside the reactor and the bio-oil from the condenser were collected. The biogas production was calculated by the deduction of bio-oil and bio-char from sample weight. Figure 2 shows the schematic diagram of the pyrolysis set up used in this research.

#### 2.2.6. Fourier Transform Infrared (FTIR) Analysis

The biomass and bio-char sample’s functional groups were investigated by Fourier transform infrared spectroscopy manufactured by Perkin Elmer, model no. FTIR Spectrum Two. All spectrums were achieved for the wavenumber from 4000 to 500 cm^−1^ with 1 cm^−1^ step size and 8 scan rates.

## 3. Results and Discussions

### 3.1. Proximate Analysis

Moisture content (MC), volatile matters (VM), fixed carbon (FC), and ash contents (AC) were analyzed by proximate analysis. These are the chemical characteristics of the biomass. Table 1 shows the proximate analysis of *Acacia Holosericea* sample. 

The volume of water present in the dry biomass sample is defined as moisture contents of that sample. If the MC values are high, it affects the reactivity of the sample during pyrolysis and reduces the quality of the biofuel [24]. If the values are less than 10%, it is effective for thermochemical decomposition to generate biofuels in the pyrolysis procedure. As the value of MC in this study is 9.56%, it is appropriate for the production of bio-char, bio-oil, and biogas [25]. For *Acacia Mangium* and *Acacia Auriculiformis,* the MC for the upper, middle, and bottom part of the trunk was found to be 12.6, 10.8, 13.6 and 13, 11.2, 13.3%, respectively, which is comparable with the values of Marsoem et al. [26]. The tree’s location, age, and sample preparation method, may be the causes for small differences. 

The Volatile Matter (VM) mostly consists of hydrocarbons, carbon monoxide (CO) gas, carbon dioxide (CO_2_) gas, and hydrogen (H_2_) gas with tars. If the values of VM are high, the reactivity will be high for that biomass sample for any thermochemical process. Normally, the values vary between 60% to 85% for any biomass sample [27]. Volatile matter for the *Acacia Holosericea* trunk was reported as 65.32 wt%. This value is comparable with other studies done for *Acacia Mangium* and *Acacia Auriculiformis* which are 64.4, 66.0, 65.4 and 64.8, 65.7, 65.6 for the bottom, middle, and top part of stem, respectively [26]. 

The ash content (AC) is a non-combustible substance that has an inverse effect in the combustion procedure. If the value of AC is low, it has high efficiency in the pyrolysis procedure [28]. The value of AC in this study for the *Acacia Holosericea* sample is 3.91% which is lower than the value of *Acacia Nilotica*, 7.3% [29]. Harvesting process and soil condition of the trees could be the possible causes of this variation [30]. Sometimes, ash content shows catalytic activity, which has an influence on the quality of the product for the pyrolysis procedure [24,31].

The percentages of fixed carbon (FC) for this biomass sample, 21.21%, was achieved by the subtraction method. The low fixed carbon content found in several species reflects their high VM, and showed that the bulk of the fuel/wood material is consumed in the gaseous condition during combustion where higher fixed carbon content attests to the high-energy value of the plant material [32].

### 3.2. Ultimate Analysis

In Table 2, the ultimate analysis of *Acacia Holosericea* is given. The atomic ratios of H/C and O/C are also provided. The value achieved for this sample is 44.03%, 5.67%, and 0.25% for carbon (C), hydrogen (H), and nitrogen (N), respectively. The percentage of oxygen is 50.05% obtained from the deduction of above values from 100.

For any biomass where the wood will be used as heating fuel, the carbon (C) content is the most significant component [33]. If the percentage of carbon is high and the value of oxygen is low, the heating value of the biomass will be high [34]. Hydrogen content of about 5.67%, nitrogen less than 1%, and no sulfur (0%) were found in this biomass feedstock. Nitrogen (N) and sulfur (S) are the most crucial for the environment because these are the main source of NOx and SOx creation. Acacia trees grow faster even in poor soil because of their nitrogen-adding capabilities [35]. As there is no sulfur present in the *Acacia Holosericea* sample, it will provide great advantages for biofuel production. The values observed in this study from the ultimate analysis for *Acacia Holosericea* samples are better than the values received from literatures for energy creation from biomass [32,36,37,38,39].

#### Van Krevelen Diagram 

**Figure 3:**
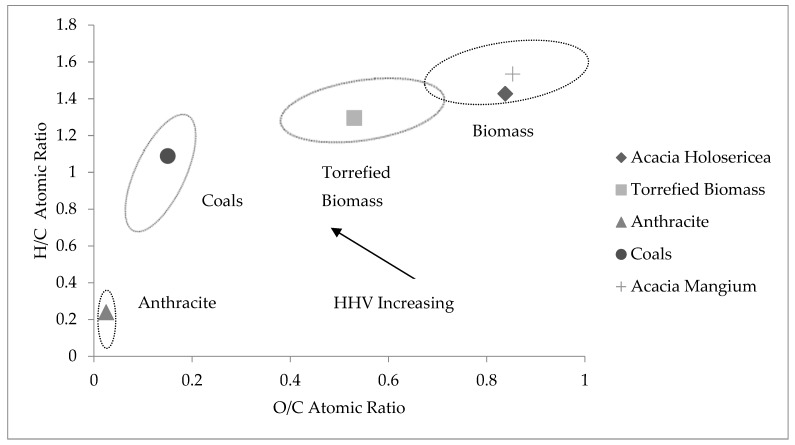
Van Krevelen graph for different samples including *Acacia Holosericea*.

In the Van Krevelen diagram, the graph was plotted for H/C atomic ratio versus O/C atomic ratio in Figure 3. The samples, *Acacia Holosericea* tree (this study), *Acacia Mangium* [40], torrefied biomass produced at 300 °C [41], Indonesian origin coals, and anthracite [42] were used for this graph. The O/C and H/C values of *Acacia Holosericea* lie on the biomass arena perfectly in Van Krevelen diagram. So it can be concluded that *Acacia Holosericea* is one of the best sources of biomass and it has the potential to produce bioenergy.

### 3.3. Scanning Electron Microscope (SEM)

**Figure 4:**
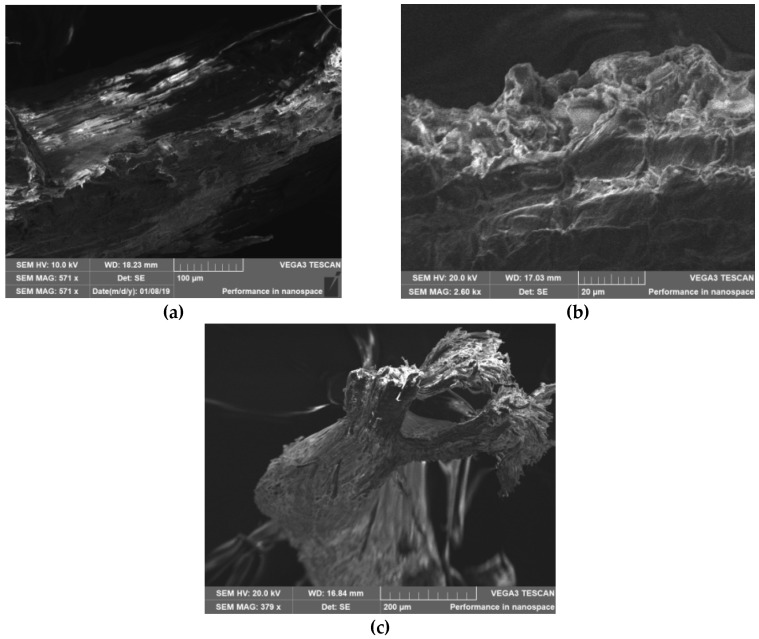
Scanning Electron Microscope (SEM) image of *Acacia Holosericea* Sample ((**a**) 200 µm, (**b**) 100 µm and (**c**) 20 µm).

Figure 4 shows the SEM micrographs of *Acacia Holosericea* sample in different scales. The sample revealed a definite shape with poorly defined pores. This image shows many fibrous structures together with a minor fraction of irregular-shaped small particles. In addition, the surface of the particles seemed to be rough probably due to the chemical compositions [18].

### 3.4. Calorific Value (Higher Heating Value)

Higher heating values (HHVs) or Calorific value is one of the most important techniques for justifying the capability of the biomass as a source of energy [43]. In Table 3, the HHV of the *Acacia Holosericea* sample has been mentioned to be 18.13 MJ/kg. The HHV in this species is greater than the results found for *Acacia Auriculiformis* and *Acacia Mangium* which were 16.8, 17.4, 17.2 and 16.6, 17.5, 17.0 MJ/kg for the upper, middle, and lower portion of the trunk, respectively [26]. HHVs result for this work is comparable with the results found for the chestnut tree, eucalyptus barks, hazelnuts, and cypress fruits [44]. 

### 3.5. Product Yield 

Pyrolysis is defined as the cleavage to smaller molecules by thermal energy. The traditional pyrolysis of biomass is connected with the product of high charcoal content, but the fast pyrolysis is linked with the products of interest, such as tar, at low temperature (675–775 K) [45], and/or gas at high temperature [46]. 

Table 4 shows the yields of bio-char, bio-oil, and biogas for *Acacia Holosericea* for two different temperatures (500 to 600 °C) with two different heating rates (5 °C/min to 10 °C/min). This result indicates that the invasive plants can be used as feedstock materials to produce value-added biofuels. The bio-char production rate decreased from 34.35% to 25.81%, when the pyrolysis temperature increased from 500 to 600 °C [47]. Ultimate analysis results indicate that the contents of carbon increase with pyrolysis temperature while hydrogen and oxygen content decrease [48]. Presence of lignin in the biomass may provide higher yields of charcoal and tar from wood [49]. The char formation from lignin under a mild reaction environment is a result of the breaking of the moderately weak bonds and the formation of more resistant dense structures [50]. One further parameter which could affect char formation is moisture content. The presence of moisture increased the yield of char from the pyrolysis of wood waste at temperatures between 660 and 730 K which is related to this study [51]. The stronger outcome of the heating rate on the formation of bio-char from biomass than from coal may be accredited to the cellulose content of the biomass [52]. It also proves that heating rate has a much greater outcome on the pyrolysis of biomass than on that of coal. The fast de-volatilization of the biomass in rapid pyrolysis favors the creation of char with high porosity and high reactivity [53]. The effect is a decrease in the volatile fuel creation and an increased yield of bio-char cellulose transformed to levoglucosan at temperatures above 535 kelvin [54].

The bio-oil yields of the feedstock are much higher at 500 and 600 °C with a higher heating rate, about 15%. This is consistent with the findings in literature studies, which showed that oil yield is highest at reasonable temperatures [55]. *Acacia Holosericea* has shown similar production efficiency for bio-oil, confirming their potential as biomass for value-added bioenergy [56]. The term bio-oil is used mainly to refer to liquid fuels. There are several reasons for bio-oils to be considered as relevant technologies, e.g., for energy security reasons, environmental concerns, foreign exchange savings, and socioeconomic issues related to the rural sector [57]. These bio-oils can substitute usual fuels in vehicle engines either totally or partially [58]. Upgrading by lowering the oxygen content and removing alkalis of hydrogenation and catalytic cracking of the oil may be required for specific applications [46]. Pyrolysis produces fuel energy with high fuel-to-feed ratios, making it the most suitable process for biomass conversion and most competitive and my eventually replace non-renewable fossil fuels [59]. Literature shows that the conversion of biomass to bio-oil can acquire an efficiency of up to 70% for the pyrolysis processes [60]. 

The syngas yields of all the samples were much more variable than the oil and char yields as it can be used directly as energy. An invasive plant can be used as a feedstock for syngas production through low-temperature pyrolysis. Syngas yield is proportional to temperature, which is comparable with our results; it increased around 10% for the temperature increase from 500 to 600 °C and the major portion will be hydrogen combined gases [61]. However, the syngas yield results indicate that the invasive plant can be utilized as the source of biogas production. The thermochemical decomposition process, such as pyrolysis, gasification, and steam gasification, is available for converting the biomass to more useful energy. Hydrogen has the potential to solve two major energy problems: reducing dependence on petroleum and reducing pollution and greenhouse gas emissions [57]. Hydrogen is not a primary fuel. It can be burned to produce heat or passed through a fuel cell to produce electricity. Hydrogen can be produced economically from woody biomass and can be thermally processed through pyrolysis [45]. Hydrogen gas has a very high potential if used in an electricity generating fuel cell. Fuel cells have no moving parts, produce only clean water as emissions, and are around 70% efficient. Hydrogen can be produced by gasification of biomass [62]. Hydrogen production from biomass requires multiple reaction steps: for the production of high purity hydrogen, the reforming of fuels is followed by two water gas-shift reaction steps, a final carbon monoxide purification, and carbon dioxide removal [19]. 

### 3.6. Fourier Transforms Infrared (FTIR) Spectroscopy 

**Figure 5:**
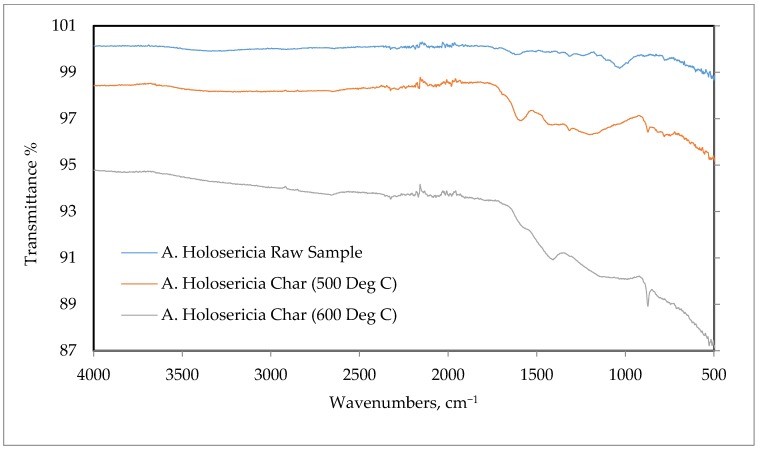
*Acacia Holosericea*, Fourier transform infrared (FTIR) spectra of biomass and bio-char for 600 °C and 700 °C.

For determining the functional group of the *Acacia Holosericea* sample, Fourier transform infrared (FTIR) spectroscopy was performed for the wavenumber of 4000 to 500 cm^−1^. In Figure 5, the FTIR analysis plots are shown, and in Table 5 the functional groups are described as per the literature standards [59]. From the result, it was found that the values of this sample are very similar to the characteristics of other biomasses. The transmittance proportion was found for the precursor and the bio-chars at 500 °C, 600 °C, are equivalent to the stretching shape of O–H grouping. The observed peaks were in the wavenumbers of 2960 to 2850 cm^−1^ was for C–H bond stretching, which is a sign of availability of cellulose, hemicellulose, and lignin in the biomass [63]. Peaks for the area 2150 cm^−1^ wavenumber are for C=C=O stretching ketene [63]. The peaks in the region of 2000–1650 cm^−1^ are for C–H bending in cellulose and hemicelluloses. The peaks in the area of 1750 and 1630 cm^−1^ are for stretching of C=O bonding in hemicellulose. The aromatic ring for C=C stretching and bending of H–O–H is for water [64]. The peaks appearing between 1384 to 1346 cm^−1^ represent the deformation of the C-H group. The significant peaks of the sample have occurred for the range of 895 and 885 cm^−1^ wavenumber. For the aromatic ring, the C=C bond stretching happened in alkene in the values of 842 to 720 cm^−1^ [59].

### 3.7. Thermogravimetric Analysis (TGA) and Derivative Thermogravimetry (DTG)

**Figure 6:**
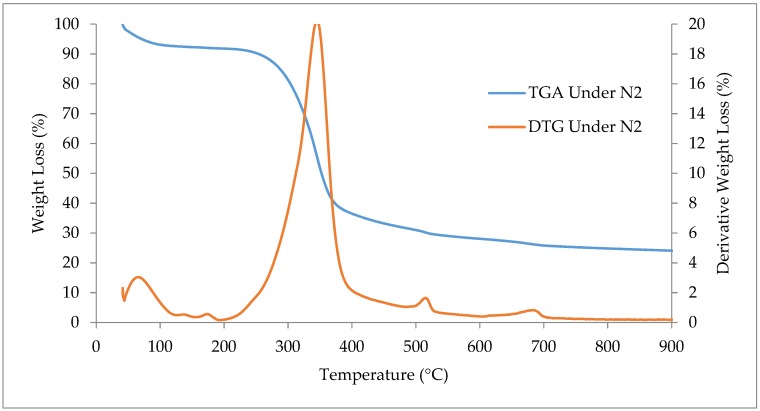
The curve of the thermogravimetric analysis (TGA) and derivative thermogravimetry (DTG) for *Acacia Holosericea* sample under pyrolysis (N_2_) situation.

The Thermogravimetric analysis (TGA) and their differential thermal (DTG) analysis were examined to determine the thermochemical behavior of the biomass under pyrolysis and combustion situations. The heating temperature was 40 °C to 900 °C with a 25 °C/min heating rate. Continuous pure nitrogen (N_2_) and pure oxygen (O_2_) gas flow was maintained for ensuring an effective atmosphere in the heating chamber. The curves of TGA and DTG under the pyrolysis situation for *Acacia Holosericea* are shown in Figure 6. Three major stages are revealed for decomposition of the biomass that are similar to Zhihua Chen et al. [65]. This is because of the loss of moisture content and the breaking of cellulose, hemicellulose, and lignin components [66,67]. Thermal degradation stages in pyrolysis situation and their region of temperature were observed for weight loss is given in Table 6. The very first decomposition was in the temperature level of 40 °C to 197 °C and was for the removal of moisture available in the sample.

Stage 2 was recorded up to 197 to 432 °C for the sample, showing the elimination of extractable composites available in the sample [68]. The main decomposition which occurred in this stage is 52.31% for the breakdown of the cellulose and hemicellulose portion [69]. The percentage of weight loss in this stage was comparable with the results of R. Zanzi-vigouroux and L. Aguiar-trujillo [70]. In the second phase, the peak decomposition temperature was traced in the DTG curve at 357 °C. 

The results achieved in this work, are very comparable with the decomposition style of pine woods because the de-volatilization process is related to the presence of cellulose, hemicellulose, and lignin of the biomass [63]. In the second stage, the highest conversion of biomass to bioenergy was made as the cellulose and hemicellulose were broken in this level. The decomposition pattern of lignin is continuous, slow, and complex [65]. In the third phase, the long tail was found for the decomposing of the biomass from a temperature of 432 °C to 900 °C. The minor rate of losses in this phase are shown; the steady and constant decomposition of lignin with the char production of non-volatile parts. The percentages of mass losses in the third phase were 13.12% which is the lowest, representing slow and constant decomposition of composite compounds. The residual or char was found 24.71% after 900 °C temperature. The peak represents the decomposition of cellulose, whereas, the tail indicates the pyrolysis of lignin [65].

**Figure 7:**
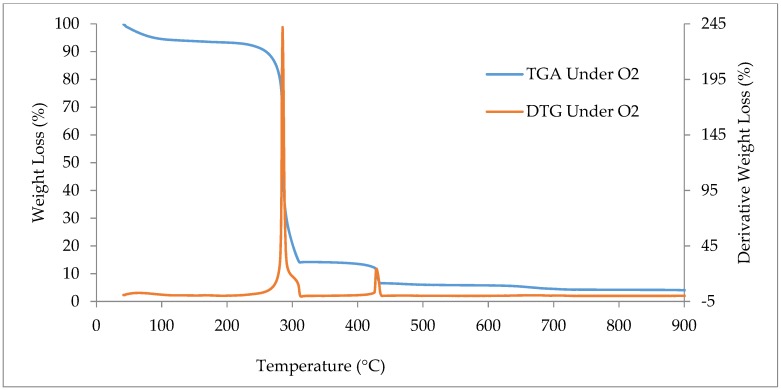
The curves of TGA and DTG for *Acacia Holosericea* in Combustion (O_2_) situation.

In Figure 7, the TGA and DTG curves of the *Acacia Holosericea* sample are shown for the combustion state. A nonstop flow of pure oxygen (O_2_) gas was supplied to the chamber for obtaining the entire combustion of the biomass sample with a 25 °C/min heating rate. The peak found at 287 °C represented the weight loss in the combustion stage, for the decomposition of lignin and volatile matter of the biomass. Due to the presence of hemicelluloses and cellulose particles in the biomass sample, major decomposition happened at the temperature of 202 °C to 437 °C. Table 7 presents the highest peak in the DTG curve for the maximum amounts of volatile matter was present there. The results achieved in this work are very much comparable with the values found in agricultural waste of Africa where it is shown that 70% to 80% of the sample weight will decompose within 600 °C [71]. The decomposition pattern has proven that this biomass can be used as a proper feedstock for producing biofuels by thermochemical degradation process [56]. 

## 4. Conclusions

Among recent advances in developing cellulosic and non-cellulosic biofuel sources, invasive plant species have proven their potential as a new area with corn, Switch grass, and others. The addition of invasive plant species as a bioenergy source will help to diversify the nation’s energy dependence and help in the reduction of the negative environmental and social impacts by energy crop production. The results of this study demonstrate that *Acacia Holosericea* is an excellent source of biomass for its rapid growth rate and easy accumulation. Furthermore, the use of these invasive plants as a biofuel does not have any direct impact on the environment. The pyrolysis experiment has proven as an outstanding tool for the thermal conversion of biomass into bio-fuel as it an easy, simple, and cost-effective process. The production range of bio-char, bio-oil and biogas was found to be 25.81~34.45%, 32.56~37.61%, 33.09~36.58%, respectively, which indicates that it is an effective source for bioenergy. There was a strong relationship between the production of bio-char, bio-oil, and biogas and heating temperature and heating rate. From TGA and ultimate analysis it was found that the moisture content was less than 10% and ash content was less than 4% which is highly recommended for any biofuel production from biomass. Through FTIR, the strong bondage between carbon, hydrogen, and oxygen established the potentiality of bioenergy. High levels of macronutrients and micronutrients in bio-chars, indicated the efficient application in water and air purification and soil management. Below ground carbon stores may provide an opportunity to reduce the impacts associated with global climate change. Due to the higher percentages of oxygenated compounds in biomass, further upgrading will be required for bio-oil for the use in a combustion engine. The decent calorific value marked the biomass as suitable for direct energy applications. Syngas yield indicated that the major portion will be hydrogen combined gases.

In future, one should focus on sample preparation as it is time-consuming. The bio-char from this biomass can be used as a major source of activated carbon for the treatment of gas and water. The characteristic of bio-oil and biogas can be examined to use it as a fuel directly or indirectly. As the *Acacia Holosericea* species has been able to successfully establish itself in degraded habitats in Brunei Darussalam and exhibit high biomass production, it could potentially help Brunei Darussalam to achieve the aims of CO_2_ emissions reduction while ensuring a positive impact on the country’s biodiversity. 

## Figures and Tables

**Table 1:** Results of Proximate Analysis for *Acacia Holosericea* sample.

Species	Moisture Content (MC)	Volatile Matter (VM)	Fixed Carbon (FC)	Ash Content (AC)
*Acacia Holosericea*	9.56%	65.32%	21.21%	3.91%

**Table 2:** Ultimate Analysis *Acacia Holosericea*.

Sample	Carbon	Hydrogen	Nitrogen	Sulfur	Oxygen ^1^	H/C	O/C
Trunk	44.03%	5.67%	0.25%	ND ^2^	50.05%	1.534	0.853

^1^ Calculated from deduction; ^2^ Not Detected.

**Table 3:** Calorific value (HHVs) of *Acacia Holosericea* trunk.

Species	Part	HHV Value	Unit
*Acacia Holosericea*	Trunk	18.13	MJ/Kg

**Table 4:** Production yield of *Acacia Holosericea* trunk.

Species	Part	Temperature (°C)	Bio-char	Bio-oil	Biogas ^1^
*Acacia Holosericea*	Trunk	500 (5 °C/min)	34.35%	32.56%	33.09%
*Acacia Holosericea*	Trunk	600 (10 °C/min)	25.81%	37.61%	36.58%

^1^ Calculated from Deduction.

**Table 5:** List of the functional groups of Fourier transform infrared (FTIR) spectroscopy for biomass and bio-char at 500 °C and 600 °C of *Acacia Holosericea*.

Functional Group	Wave Number (cm^−1^)
O–H stretching in cellulose and lignin	3600–3000
C–H stretching in aliphatic formation	2960–2850
C=C=O stretching ketene	2150
C–H Bending in cellulose and hemicellulose	2000–1650
C = O stretching of hemicelluloses	1750–1630
C–H deformation in cellulose and hemicellulose	1384–1346
C–O stretching vibration in cellulose and hemicelluloses	1085–1050
C=C stretching alkene vinylidene	895–885
Aromatic rings	842–720

**Table 6:** Different stages of the thermogravimetric analysis (TGA) and derivative thermogravimetry (DTG) for *Acacia Holosericea* in pyrolysis (N_2_) environment with weight loss.

Item	Stage (1)	Stage (2)	Stage (3)	Remark
Temperature (°C)	40–197	197–432	432–900	357 (Peak)
Weight Loss (%)	9.86	52.31	13.12	24.71 (Residue)

**Table 7:** Different stages of the TGA and DTG for *Acacia Holosericea* in combustion (O_2_) environment with weight losses.

Item	Stage (1)	Stage (2)	Stage (3)	Remark
Temperature (°C)	40–202	202–437	437–900	287 (Peak)
Weight Loss (%)	9.86	81.93	4.23	3.98 (Residue)

## Supplementary Materials

The following are available online at https://www.mdpi.com/2306-5354/6/2/33/s1, **Figure S1.** Distribution map of Acacia Species around the world [72]. **Figure S2.** *Acacia Holosericea* in Brunei Darussalam. **Figure S3.** Flow diagram of *Acacia Holosericea* sample preparation.
