# Supplementary material for: Acacia Holosericea: An Invasive Species for Bio-char, Bio-oil, and Biogas Production
Source: Bioengineering (Basel). 2019 Apr 16;6(2):33. doi: 10.3390/bioengineering6020033 (PMC6630911; doi:10.3390/bioengineering6020033)
Supplement: Supplementary file 1 [file bioengineering-06-00033-s001.pdf]

Supplementary Materials:

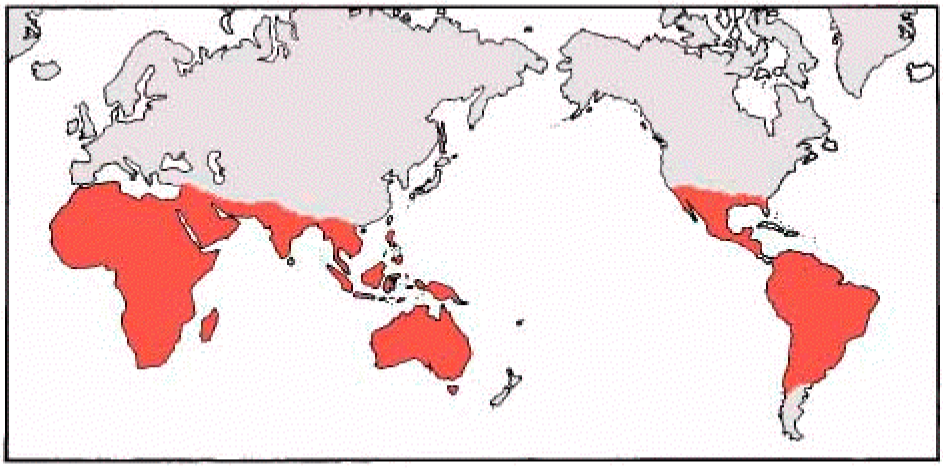

Figure S1. Distribution map of Acacia Species around the world [74].

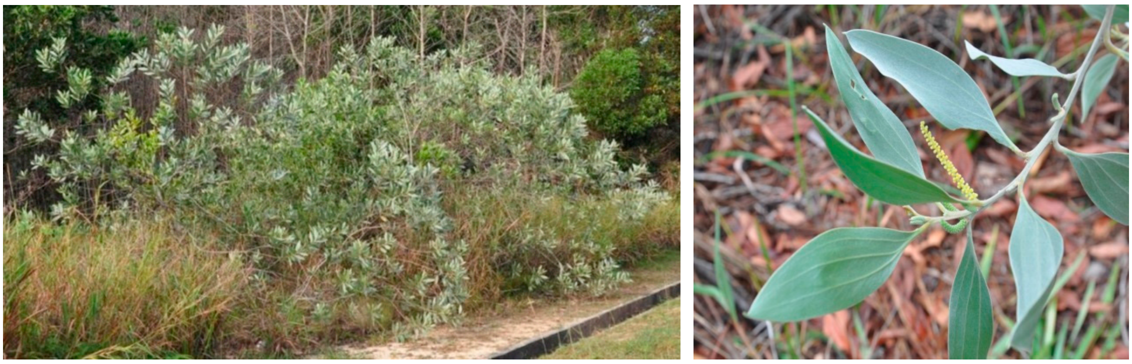

Figure S2. *Acacia Holosericea* in Brunei Darussalam.

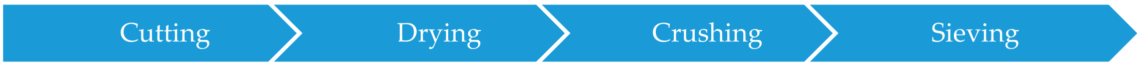

Figure S3. Flow diagram of *Acacia Holosericea* sample preparation.
